# An approach for unplanned dilution assessment in open stope with consideration of the stope shape irregularity

**DOI:** 10.1038/s41598-025-18111-w

**Published:** 2025-09-29

**Authors:** Aibek Alipov, Amoussou Coffi Adoko

**Affiliations:** 1 School of Mining and Geosciences, Nazarbayev University, Astana, 010000 Kazakhstan; 2 Kazzinc Corporation, Ust-Kamenogorsk, 070002 Kazakhstan

**Keywords:** Unplanned dilution, Rock engineering system, Numerical modeling, Dilution graph method, Equivalent linear overbreak/Slough (ELOS), Natural hazards, Engineering

## Abstract

This study aims to propose an assessment tool for unplanned dilution, considering the irregularity of the stope shapes due to the overbreak and underbreak of the stope walls. Data from the Ridder-Sokolny mine, an underground mine, located in East Kazakhstan, was used for the study. On the basis of the collected dilution data, the stopes were categorized into three types depending on the extent of the irregularity: simple, semi-complex, and complex shape. Numerical modelling was performed to illustrate the effect of the stope shape complexity on the overall instability, while the Rock Engineering System (RES) was employed to introduce a Dilution Index (DI) with the purpose of quantifying the stope shape irregularity effect. The results suggested that instabilities and rock failure are likely to occur with an increase in the stope shape complexity as tension increases while the strength factor decreases. Furthermore, the stope shape irregularity had the most significant effect on the DI system compared to other parameters affecting the dilution. The results indicated that the DI is a very good predictor of unplanned dilution and a fairly good predictor of stope loss, which indicates advantages over the conventional dilution graph method. It is concluded that the DI could be an alternative for unplanned stope dilution estimations depending on the stope shape complexity.

## Introduction

In underground mining operations, it is of vital importance to reduce production costs, minimize unplanned dilution, and improve production and safety. One of the most crucial steps toward these goals is the optimal design of the stopes. As mining progresses deeper, the likelihood of having poor stope design performances, such as stope wall stability and dilution, generally increases. Dilution is defined as the proportion of waste rock that has to be mucked with ore during the mining process and is related to mining efficiency^1^. It is generally categorized into 2 types: planned dilution (it is quite predictable since it is due to mining development, geologic contacts or resource estimations); and unplanned dilution^2^. While the planned dilution (also known as primary dilution) is quite predictable, unplanned dilution estimation, on the other hand, is very challenging since it is commonly associated with overbreak and wall sloughing as a result of poor blasting, weak rock, or inadequate stope. Urli and Esmaieli^3^ noted that stope hangingwall sloughage is one of the major sources of unplanned dilution in open stope underground mines. Unplanned dilution poses economic challenges to mining companies worldwide. It has a direct and significant effect on the cost of each underground stope. Thus, it is advantageous to establish a quantitative correlation between the dilution and its influencing parameters, as it can be used to improve the ground safety, the production capacity and enhance the overall profitability by minimizing unplanned ore dilution^4^.

Driven by these motivations, numerous authors have investigated extensively research topics related to unplanned dilution and sloughing over the past few decades^4–9^. It has long been recognized that the stope geometry (including stope shape, size, dimensions, and inclination) is one of the key parameters influencing the level of unplanned dilution in open stope mining^10–14^in addition to other factors such as the induced in-situ stresses, the blasting patterns, the surrounding rock mass properties, and the operational requirements. This has been sufficiently substantiated in the literature, revealing a wide range of results obtained using analytical, empirical, and numerical methods, usually as a combination of any of these, which represent the general design approach for any mine structure^10^. For instance, one of the earliest studies on the effects of geometry on the stability of an underground excavation can be traced back to when Terzaghi^15^ quantified the influence of a tunnel’s height and width on its stability. Another significant early study was the introduction of the concept of an unsupported span and its equivalent stand-up time by Lauffer^16^. Years later, the concept of span or width, hydraulic radius or radius factor of an underground excavation were used through rock mass rating systems^17–19^and the empirical stability graph method^12,20,21^ to account for the effect of the excavation geometry on its stability.

In the stability graph method, the hydraulic radius of the individual stope surfaces is related to the stope shapes that are limited to the simple excavations of a rectangular cross-section. However, as practice shows, the shape of the stopes in real-life scenarios does not always obey the original profile, and often it is much more complicated due to various factors. To tackle this complexity, a radius factor has been suggested as a replacement for the hydraulic radius^22^while an index for stope geometry (by dividing the volume of a stope by its real surface area) was defined as a measure of stope geometrical complexity^23^. Similarly, with the purpose of considering the effect of stope geometry on dilution and sloughing, other key studies were accomplished. For example, Clark^5^ used the concept of the equivalent linear overbreak or slough (ELOS) parameter to evaluate the amount of dilution of a stope hanging wall while other authors showed that the occurrence of strainburst in highly stressed ground can be due to the geometry of excavations^24–26^. Unplanned dilution related to stress relaxation has also been attributed to stope geometry, and the effects of stope geometries were described and correlated to the stress relaxation and dilution using numerical modeling and dilution-based design graphs^27,28^. Henning and Mitri^29^ suggested that geometry complexity is a critical factor influencing the overbreak potential, and the stope height effect and dip angle of hanging wall have a significant effect on dilution compared to other parameters. Capes^13^ demonstrated the critical role of stope wall geometry in the design of open stope in unfavorable conditions. In a narrow vein mining environment, it has been demonstrated that the stope strike length, ore dip, and the narrow nature of the stope have a major influence on overbreak and dilution in different ways^11,30,31^.

More recently, the effect of 3D stress on stope design was correlated to the shape factor in terms of stope wall and back lengths through numerical modeling and statistical approach^32,33^. Equally, the stope geometry was considered an important parameter in the establishment of a stability economy model for an open stope in order to minimize dilution within the context of the ore-skin design^3^. Furthermore, a recent study discussed the evaluation of the effect of stope geometrical parameters on the brittle damage of rock mass. Through the so called response surface methodology, the effects (individual and interactive) of input parameters on response variables were quantified, and the increased values of geometrical parameters had shown higher influence on rock mass brittle damage^34^.

Despite the fact that stope geometry has been extensively studied, there has been less effort dedicated to the effect of stope shape irregularity (i.e., the overall shape of cross sections resulting from the stope surface irregularities) on the unplanned dilution, and only the width or span has been considered as the most representative of stope geometry^23,35^. Yet the problem of shape irregularity was pointed out by many authors^29^. However, in practice, the design of stopes does not necessarily feature a complex or irregular geometry. This irregularity may result from poor blasting, geological setting, and operational constraints. This leads the cross section to having more sides, edges, and vertices, which allows for tension accumulation, eventually causing spalling and slabbing failure^36^. This surface complexity increases the likelihood of structure-driven instabilities due to the orientation angle between the discontinuities and the stope surface. The combined action of these aspects results in an even redistribution of stress around the stope and relaxation-related dilution^37,38^. Therefore, the stope surface character is another important factor in controlling unplanned dilution and overall stability.

Due to the limitations of the existing stope design practice as mentioned earlier, this current study aims to quantitatively assess the effect of stope shape irregularity on unplanned stope dilution. To this end, a methodology using numerical modeling and the rock engineering system (RES) is implemented. The numerical simulations are used to illustrate the effect of the stope shape complexity on the overall instability. Numerical modeling is commonly used in mine stope design for stress-strain analysis around excavations^39–41^. Meanwhile, the RES approach, via an interaction matrix, is employed to define a new Dilution Index (DI) capable of quantifying the stope shape irregularity effect. The proposed methodology is motivated by several existing studies in which the RES has been proven to be an adequate tool for quantifying the rock mass behavior around underground excavation^42–45^. More recently, Zhang, et al.^46^ successfully proposed causality-based RES for risk assessment and prediction of rock fragmentation in blasting operations in surface mines. Their study highlighted the superiority of the RES-based model over existing models in terms of its ability to predict rock fragmentation. Other recent applications of the RES methodology vary broadly and include stability analysis of bench berms in open pit mining^47^; abandoned mine instability assessment with case studies^48^; prediction of face advance rate and determination of the operation efficiency in retreat longwall mining panel^49^ and prediction of rotary drilling penetration rate in iron ore oxide rocks^50^. These justify the adopted methodology. To develop the DI, a database consisting of the stope dilution parameters is compiled based on field data collected from the Ridder-Sokolny mine located in East Kazakhstan.

## Study area and data description

The Ridder-Sokolny mine is located in the mountainous area of East Kazakhstan, approximately 3 km from Ridder city. With more than 200 years of history, Ridder-Sokolny mine is owned by Kazzinc Corporation and currently produces 1.6 million tons of ore per year with an average gold grade of 2.0 g/ton^51^. The Ridder-Sokolny polymetallic deposit pertains to the Leninogorsk ore field within the W–E striking regional Semipalatinsk–Leninogorsk fault with a geological structure described as weakly deformed layered strata of the arch of anticlinal structure^52^. The bulk of industrial mineralization is concentrated in the rocks of the Krukovka formation. In general, the ore deposits are covered by volcanic-sedimentary Devonian sediments (a layer of lavas, tuffs of andesite-basalt composition, tuffaceous conglomerates and sandstones (100 m thick), above which a bundle of argillite and aleurolite with the lenticular bodies of rhyolite extrusive facies with a thickness of more than 400 m is deposited^53^. The Ridder-Sokolny deposit consists of a series of ore deposits. The vast majority of ore bodies are of small thickness with variable length, both along strike and dip. The main ones are Pobeda, Central, 1st, 2nd, 3rd Southwestern, and Bystrushinskaya. In general, ore bodies have a complex shape with the vertical span of the ore zone exceeding 600 m. The main ore minerals are sphalerite, chalcopyrite, galena, and pyrite^54^. Several mining methods are utilized at the Ridder-Sokolny mine depending on ore body morphology and thickness, such as sublevel caving, cut-and-fill stoping, sublevel stoping with partial shrinkage applied, shrinkage stoping, upward horizontal slicing with backfilling, and sublevel caving^53^. The main products after processing include gold, copper, zinc, and lead concentrates.

The rock mass of the area of study consists mainly of six geotechnical domains with variable mechanical properties, from very stable to unstable ground. The rock domains were selected based on the occurrence of the minerals at the particular location. The strongest rocks consist of microquartzites, agglomerate tuffites, and the weak ones are sericite-chlorite-quartz rocks and schists. Significant variability (variation coefficients 25–35%) of the rock strength is noted even within the same type of rock. Table 1 shows the geomechanical parameters of the rockmass in the Ridder-Sokolny mine. Field mapping reveals the occurrence of four main sets of joints within the ore deposit areas. The joint spacing and the orientation ranges are shown in Table 2. The first three systems of joints are almost mutually perpendicular to each other and form a block structure of the rockmass, close to cubic blocks.

In order to monitor the effect of the mined-out stopes on the overall stability of the mine (for long-term development) and to estimate the total dilution, the stopes were systemically surveyed in the Ridder-Sokolny mine using a cavity monitoring system (CMS), the “Void Scanner 150 Mk3”. The surveyed stopes were reconciled with the planned stopes to measure the actual dilution and loss. Using these data together with the mine planning, stope design, and geological data, a dilution database was established.

**Table 1:** Rock domain properties.

Rock domain		Rock mass					Intact rock		
Lithology #	Main rock types	Q’ value	RMR	RQD	GSI	Density (t/m3)	UCS (MPa)	Young mod. E_i_ (GPa)	Poisson ratio, ν
#1	Tuffs, felsite-porphyry, and porphyrites	20	68	74	75	2.74	135	69	0.19
#2	Micro quartzite and sericitic microquartzite	15	68	58	61	2.72	158	73	0.2
#3	Aleuropelites, quartzite albitofir, tuffs, and ores	16	58	61	54	2.71	116	73	0.19
#4	sericite and shale	9	54	34	48	2.72	56	70	0.19
#5	sericite and carbonate rocks	17	60	62	57	2.79	27	66	0.19
#6	Sericite, chlorite and quartzite rocks	19	60	71	57	2.68	82	61	0.15

**Table 2:** Orientation of the joint sets in Ridder-Sokolny polymetallic deposit.

Parameters	Orientation		Spacing
Sets	Dip °	DipDir, °	Mean m
Joint 1	0–45	0-130	0.5
Joint 2	75–90	155–185	0.5
Joint 3	65–90	65–90	0.6
Joint 4	75–90	100–130	0.7

## A brief overview of the empirical design method of open Stopes

Empirical tools for stope design have gained popularity over the last thirty years largely due to their predictive capability and flexibility, taking advantage of the availability of stope performance data^10^. They present a practical use since conventional methods (for example. analytical and numerical modeling) of assessment have the difficulty of identifying the jointed nature of the rock material and consequently assigning design properties as input parameters for numerical simulations. The methods use the variables observed to be the most critical for design based on experience, including the rock mass quality (stability number) and opening geometry (hydraulic radius), to compare results of recorded stope performance data. The open stope design empirical methods can be divided into stability graph (qualitative) and dilution graph (quantitative) methods, in which statistical tools and, more recently, machine learning tools are often used to objectively derive design relationships^13,55^.

**Fig. 1:**
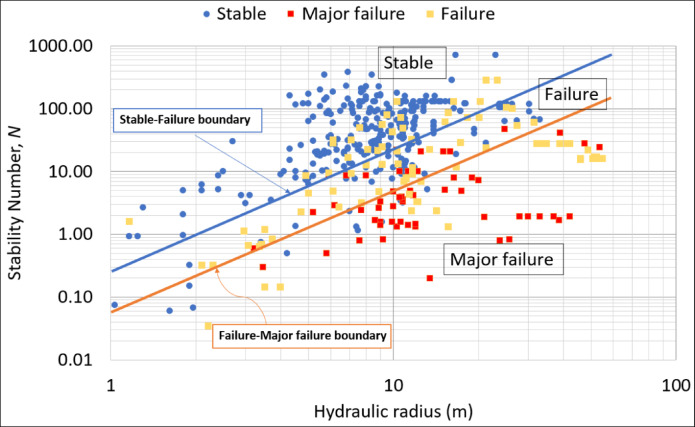
An example of the stability graph.

Over the past four decades, the stability graph method introduced by Mathews, et al.^21^ has been used at various mine sites across the world, making possible a collection of a significant amount of new data, which has allowed for a series of modifications of the stability graphs to account for a wide range of stope design conditions. As a result, the literature reveals the existence of several variants of the stability chart to date^32^. Basically, they are is a plot of the stability number N against the hydraulic radius (HR) with several stability zones. The main principle behind these charts is that for a known stability number, it can be used to determine the critical stope dimensions that would ensure desirable stability conditions. An example of a stability graph is given in Fig. 1. The graph, which was reproduced using the extended stability graph dataset compiled by Mawdesley, et al.^56^ shows 3 zones of stability conditions: stable, failure and major failure. The graph shows 3 zones of stability conditions: stable, failure, and major failure. Each zone is identified with a boundary. Roughly, the stable-failure boundary separates the stable zone from the failure zone, while the failure-major failure boundary delimits failure from major failure.

More specifically, N is defined as follows:

1 $$N=Q' \times A \times B \times C$$

where A is the rock stress factor, B is the joint orientation adjustment factor, and C is the gravity effect adjustment factor. *Q’* represents the rock mass quality and is defined as:

2 $$Q'=\frac{{RQD}}{{{J_n}}} \times \frac{{{J_r}}}{{{J_a}}}$$

In Eq. 2, the parameters are some of the well-known inputs of the Q-system for rock mass classification (Barton et al. 1974) where RQD is the rock quality designation, *J*_*n*_ is the joint set number, *J*_*r*_ is the joint roughness number, and *J*_*a*_ is the joint alteration number. Details on how to determine the factors A, B, and C in Eq. (1) can be found in^20^.

The parameter HR, which reflects the geometry of the stope faces, is defined by dividing the area of a stope face by its perimeter as follows:

3 $$HR=\frac{{{\text{Area (}}{m^2}{\text{)}}}}{{{\text{Perimeter (}}m{\text{)}}}}$$

On the other hand, the dilution graph uses the same principle to estimate the dilution in the stope walls. Taking advantage of CMS data, Clark and Pakalnis^57^ introduced the Equivalent Linear Overbreak/Slough (ELOS) concept to quantify the stope hangingwall overbreak and sloughing independently of the ore width. ELOS is defined as the overbreak volume of the rock from the stope hangingwall side divided by the area of hangingwall which gives an average depth of failure over the hangingwall surface^13^. This definition eliminates any confusion arising from the various definitions of dilution in use within the industry, depending on whether a wide or narrow orebody is exploited. This concept is more beneficial for mine planning purposes as it involves quantitative stope performance in terms of percentage of overbreak and dilution output. A dilution graph can also be established, similar to the stability graph (Fig. 1), by superimposing the dilution data points on the stability graph and then drafting in the best-fit design lines with the assistance of logistic regression with engineering judgment. In practice, these graphs are used as charts to assess the stability and the amount of unplanned dilutions for a new stope wall given the design parameters (N and HR).

## Numerical simulation

The RS2 software from Rocscience, which implements a 2D finite element analysis, was used to simulate the effect of the stope shape on the unplanned dilution. The Ridder-Sokolny orebody widths vary between small and moderate with variable length along the strike. The dimensions of the cross sections of the stopes fall within the geometric conditions for a 2D plane strain numerical model. Therefore, the RS2 software was judged good enough to simulate the shape irregularity effect. Various stope geometries were simulated (from simple shapes to very complex shapes) with variable dimensions (10 m x 40 m and 20 m x 20 m, width x height). In this study, geometry and shape basically denote the same thing and are used interchangeably herein. Table 1 displays the input material properties for the simulations. For simplicity, one rock domain (lithology #2) was used. This lithology was the most representative in the area of study. The RocData application from the Rocscience software package was also used to determine the rock mass properties. For the model meshing, 6-noded triangles (0.1 gradation factor) were used. The initial element loading consists of field stress (gravity) and body force. The mining depth was considered as 500 m and the model displacements were restrained at the boundaries. Elastic isotropic model with the generalized Hoek-Brown failure criterion was implemented. It was assumed that the sloughing around the stope was mainly due to instabilities induced by stress for simplicity. Besides, any structural-controlled sloughing due to critical discontinuities would be dealt with, in principle, on an individual basis.

The strength factor and the deviatoric stress based damage criterion are employed to determine the extent of any potential unplanned dilution. Recent research showed that stress damage is one of the key factors leading to sloughing^11^. The stress damage criterion is defined as:

4 $${\sigma _1} - {\sigma _3} \cong {\sigma _{ci}}$$

where ${\sigma _{ci}}$ is the in situ crack initiation stress. It has been suggested that the in situ crack initiation stress occurs at approximately 0.3 times the UCS^11,26^. As for the strength factor, it can be used to indicate the tension damage. In RS2 the strength factor is computed by dividing the rock strength by the induced stress at every point in the mesh. All three principal stresses are considered in the strength factor (σ_1_, σ_3_ and σ_z_), so the strength factor in RS2 is 3-dimensional.

**Fig. 2:**
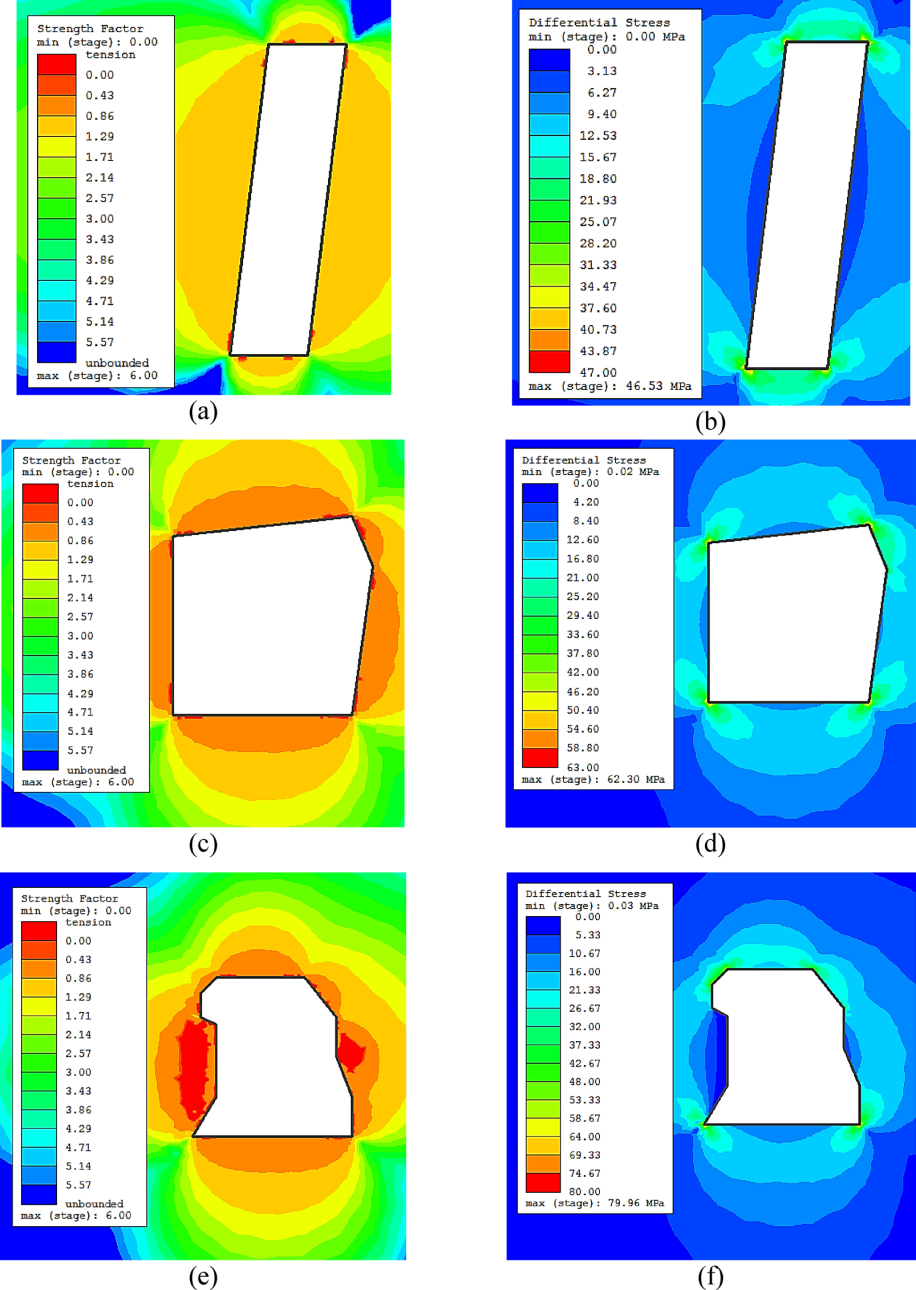

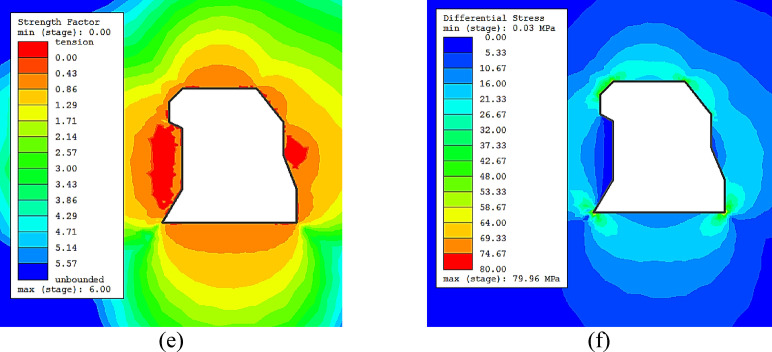
(**a**) Strength factor contours for simple shape stope 10 x 40 m, (**b**) σ1- σ3 contours for simple shape stope 10x40 m, (**c**) Strength factor contours for semi-complex shape stope 20x20 m, (**d**) σ1- σ3 contours for semi-complex shape stope 20x20 m, (**e**) Strength factor contours for complex shape stope 20x20 m, (**f**) σ1- σ3 contours for complex shape stope 20x20 m, (**g**) Strength factor contours for complex shape stope 10x40 m, (**h**) σ1- σ3 contours for complex shape stope 10x40 m.

Selected simulation results are provided in Figs. 2a-h. In general, the results indicated that the damaged zone around the stope increases with the complexity of the stope. The strength factor contours showed tension. This means σ_3_ is less than the cutoff tension specified for the failure criterion. It was observed that the extent of the tension contours increased with the complexity of the shape; this implies that the stopes are likely to experience overbreak and dilution due to tension damage. It was found that the high stress damage is not likely to occur, as the differential stress is not high enough. The value of the σ_c_ used in the analysis was 158 MPa, which corresponds to a minimum of 53 MPa of differential stress for the failure occurrence. This shows that differential stress will not significantly impact the dilution; this is mostly in agreement with field observation in the Ridder-Sokolny mine. However, rock domains with lower compressive strength could be affected by differential stress and can be exposed to stress damage, where even more dilution will occur.

## Establishing the Dilution index (DI)

### RES overview

**Fig. 3:**
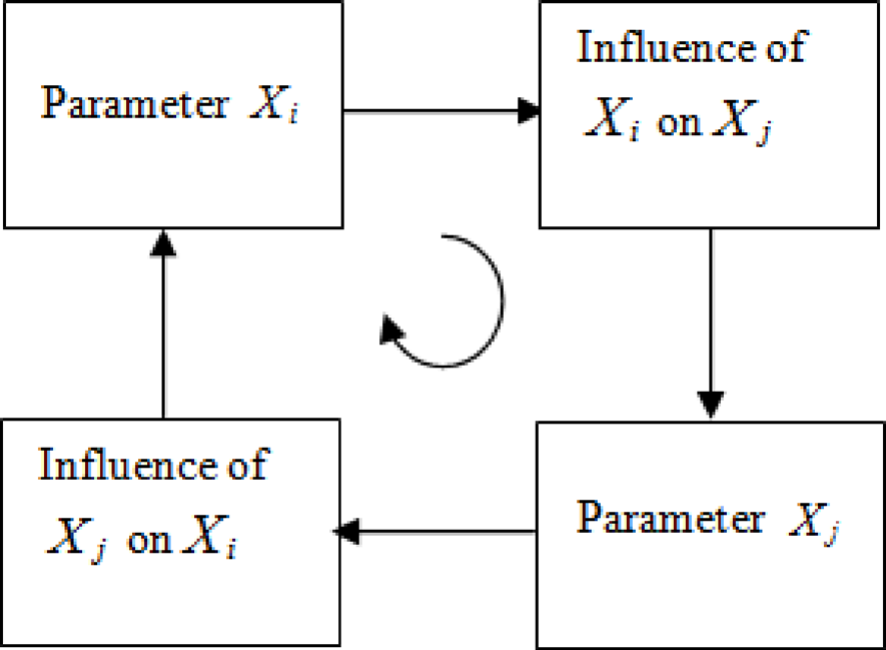
Principle of the interaction matrix.

The Rock Engineering System (RES) is a useful methodological tool that can quantify correlations among multiple components of rock engineering problems. Introduced by Hudson^42^the RES method employs a cause-effect mechanism that relates every factor of the engineering site to every other factor with different weights according to their influence. The RES working principle is illustrated in Fig. 3. The interaction matrix (IM), which reflects the mutual interrelations between pairs of input parameters, is constructed by diagonally arranging the system parameters *X*_*i*_ and *X*_*j*_. Then, using a clockwise convention, the parameters’ interactions are represented in the off-diagonal boxes. In Fig. 3, the concepts of influence and sensitivity are also illustrated. As a result, a multiple-dimensional interaction matrix containing *n* input parameters P_n_, a $n \times n$matrix, is obtained. The element situated to the right of *X*_*i*_ and above *X*_*j*_ represents the influence of *X*_*i*_ on *X*_*j*_ or the sensitivity of *X*_*i*_ toward *X*_*j*_. It should be noted that usually, the IM is asymmetric, as the influence of *X*_*i*_ on *X*_*j*_ can be different from the influence of *X*_*j*_ on *X*_*i*_ if the matrix is not symmetric.

However, because the inputs of the RES are not necessarily numerical attributes, it is often needed to assign numerical values to the interactions (off-diagonal components${I_{ij}}{\text{,(}}i \ne j)$). This step is known as the matrix encoding. After the matrix has been coded, the cause-effect interactions can be quantified. The sum of the row values for each parameter is called the cause value and denoted as *C*_*i*_, and the sum of the column values is the effect value *E*_*i*_. The parameter interaction intensity (PII) of each parameter, which is calculated by$({{C+E)} \mathord{\left/ {\vphantom {{C+E)} {\sqrt 2 }}} \right. \kern-0pt} {\sqrt 2 }}$ and the parameter dominance (PD) by ${{(C - E)} \mathord{\left/ {\vphantom {{(C - E)} {\sqrt 2 }}} \right. \kern-0pt} {\sqrt 2 }}$, can be used to recognize the degree of interaction of the parameters^42,44^.

The cause ${C_i}$and effect ${E_j}$ associated with the $ith$ parameter ${P_i}$ corresponding to the $ith$ row and the $jth$ column are defined as follows^42^:

5 $${C_i}{\text{=}}\sum\limits_{{j{\text{=}}1}}^{n} {{I_{ij}}} {\text{; }}{E_j}{\text{=}}\sum\limits_{{i{\text{=}}1}}^{n} {{I_{ij}}}$$

where ${I_{ij}}$ represents the interactions of the IM; ${C_i}$ is calculated over $jth$ column while${E_j}$ is obtained over $ith$ row. Then, the influence weights assigned to each influencing parameter can be written as the percentage of the sum of the system’s cause and effect according to the following equation:

6 $${W_i}{\text{=}}\frac{{{C_i}{\text{+}}{E_i}}}{{\sum {{C_i}{\text{+}}\sum {{E_i}} } }} \times 100$$

The dilution index (*DI*), which quantifies the dilution and overbreak associated with each stope, is computed as:

7 $$DI{\text{=}}\sum {{{({W_i})}^\beta } \times {V_i}}$$

In Eq. (6) ${V_i}$ denotes the rating values^44^ assigned to each parameter ${P_i}$ for the dilution evaluation. In the present study, the parameter$\beta$is used to “tune” the DI values and to reflect non-linear relationship between DI and its influential parameters. The values of DI range from 0 to 100. A higher value of DI implies a higher dilution.

### Parameters selection and rating

The parameters selected to establish the DI consist of the stope shape category (P1), mining depth (P2), stope hangingwall hydraulic radius (P3), modified rock mass quality index Q’ (P4), stress factor A (P5), joint orientation factor B (P6), and gravity factor C (P7). The selection of these parameters is justified by their potential influence on dilution. They are used in the empirical dilution graphs^11,13^except for P1 and P2.

The numerical simulation results indicate that P1 influences the dilution level, while P2, the mining depth, was used to account for the vertical stress. In principle, P2 and the stress factor P5 could be redundant in the DI system; however, the collinearity between these two parameters was checked, and it was found that the coefficient of linearity R was less than 0.3; hence, both parameters were kept for the development of the DI system. Besides, as P2 changes, the rock mass properties change as well due to the inherent spatial variability. A large hydraulic radius favors instability and dilution. The Q’ defined by RQD, Jn, Jv, and Ja is used to account for the rock mass quality. The stress factor A is an indicator of stress damage; lower values indicate potential instability. The stress factor A considers the effects of induced stresses and varies from 0.1 for high compressive stresses to 1 for relaxed conditions (including tension), with intermediate situations between the two extremes. The joint orientation factor B is based on the intuition that shallow-dipping joints are more likely to cause instability than steeply-dipping joints. In other words, shallow-dipping joints are more likely to be intersected by newly induced fractures caused by tensile stress conditions or blasting. Hence, acute angles of less than 20° are unfavorable to the stope wall stability. The gravity factor C accounts for the mode of failure, which may be in the form of gravity fall, sliding, or slabbing.

The interaction matrix of the DI system is obtained by rating each parameter on a scale of 0 to 4, depending on how they influence each other. The expert semi-quantitative method was used based on author experience and the available data (Hudson, 1992; Adoko et al., 2016). For example, the shape category has no influence on the depth; therefore, the influence of P1 on P2 is 0. However, depth could have an indirect influence on P1; hence, the influence of P2 on P1 is 3. Other methods do exist, such as neural network-based interaction matrix^44^. Nevertheless, in this instance preference was given to the semi-quantitative approach because of its flexibility.

For the rating of these 7 input parameters of the DI system, intervals or classes were used to discretize the domains of each attribute (numerical or logical) and each class is rated with values ranging from 0.0 to 1.0. The ratings are assigned so that higher values indicate a greater likelihood of dilution potential. More specifically, the parameters’ domains were divided into 5 intervals for all variables expect P1, which was divided into 3 intervals. Hence, the discrete values assigned were 0.2, 0.4, 0.6, 0.8, and 1.0 and 0.1, 0.5, and 1.0, respectively. For P1, being a categorial variable, the assignment of these discrete values was performed through a straightforward encoding in which 0.1, 0.5, and 1.0 corresponded to the features (simple shape, semi-complex shape and complex shape), respectively. The P6 rating is just the adjustment values of the joint orientation factor in the stability graph. For the remaining parameters, to estimate the threshold values corresponding to their physical spaces, 20, 40, 60, and 80% of their cumulative distributions were used. These values were readjusted for some cases through trial and error after checking for statistical representation as suggested by previous studies^58,59^. The physical ranges of parameters along with their corresponding ratings are provided in Table 3 while a sample of the raw data used in this study is shown in Table 4.

**Table 3:** Summary of classification categories and ratings.

Parameters	Classification categories and ratings				
Stope shape category (P1)	Simple		Semi-complex	Complex	
Rating	0.1		0.5	1.0	
Mining depth (P2), m	< 250	250–350	351–400	401–450	> 450
Rating	0.2	0.4	0.6	0.8	1.0
Hydraulic radius (P3), m	< 3	3.0–6.0	6.01-9.0	9.01-11	> 11
*Rating*	0.2	0.4	0.6	0.8	1.0
Rock mass quality Q’(P4)	< 10	10–16	16.01-20	20.01-27	> 27
*Rating*	1	0.8	0.6	0.4	0.2
Stress factor A (P5)	< 0.10	0.10–0.2	0.21–0.30	0.31–0.40	> 0.4
Rating	1	0.8	0.6	0.4	0.2
Joint orientation factor B (P6),	0°	20°	45°	60°	90°
*Rating*	0.5	1	0.8	0.3	0.1
Gravity factor C (P7)	< 5.1	5.1-6	6.01-7	7.01–7.5	> 7.5
*Rating*	1.0	0.8	0.6	0.4	0.2

**Table 4:** Sample of data used in this study.

Shape cat.	Depth (m)	HR (m)	σ_max_	Dip(°)	Dip.Dir.(°)	Q’	A	B	C	*N*’	Dilut. (%)	Loss (%)
1	480	5.23	65.92	90	170	26.67	0.30	0.24	8.00	15.36	20.08	82.10
1	420	3.84	70.42	70	235	28.89	0.30	0.20	5.95	10.31	8.05	75.00
3	390	4.27	64.50	80	150	26.66	0.45	0.30	7.00	25.19	35.08	5.21
2	365	2.18	63.35	65	250	13.33	0.30	0.20	7.47	5.98	25.02	25.19
1	380	2.10	53.12	80	240	25.00	0.30	0.20	6.96	10.44	10.01	80.07
3	390	4.27	64.50	80	150	26.66	0.42	0.20	6.96	15.58	21.06	13.16
1	480	5.23	65.92	90	170	25.00	0.14	0.24	8.00	6.94	10.04	60.21
2	365	5.01	60.16	85	245	8.00	0.11	0.20	5.60	0.99	70.05	18.15
1	480	5.23	65.92	90	170	33.78	0.22	0.24	8.00	14.27	7.06	65.22
3	450	6.00	76.00	65	190	15.47	0.11	0.20	5.46	1.84	56.01	5.15
1	420	3.25	71.17	60	250	9.00	0.10	0.20	5.20	0.94	80.03	9.21
1	420	3.25	71.17	70	250	9.00	0.12	0.20	5.95	1.34	88.91	9.76
1	420	3.25	71.17	70	250	12.00	0.10	0.20	5.95	1.43	55.06	6.22
1	480	5.23	65.92	90	170	31.11	0.14	0.24	8.00	8.64	25.08	75.20
2	341	7.47	56.33	85	170	13.33	0.19	0.20	7.48	3.80	50.02	20.18
1	420	5.55	65.26	75	220	33.33	0.30	0.29	6.44	18.95	2.09	65.16
3	200	12.24	31.00	70	140	15.47	0.45	0.20	5.95	8.25	39.17	10.01
3	360	4.57	61.84	65	55	9.00	0.16	0.20	5.46	1.57	67.78	8.48
1	455	7.90	65.14	75	155	35.56	0.30	0.20	7.20	15.36	25.04	75.14
1	329	3.26	41.72	90	230	15.47	0.30	0.30	8.00	11.18	8.06	60.19
1	455	7.90	65.14	75	155	28.89	0.15	0.20	6.45	5.51	23.27	19.17
3	360	4.57	61.84	65	55	17.33	0.54	0.20	6.00	11.23	8.06	12.02
3	424	8.99	55.71	90	165	26.66	0.11	0.22	8.00	5.17	35.04	10.38
3	360	4.57	61.84	65	55	16.00	0.55	0.20	5.46	9.62	22.01	10.13
1	365	2.10	53.12	80	240	19.73	0.40	0.22	6.96	12.08	3.00	70.17
2	365	2.18	63.35	65	250	15.47	0.16	0.20	5.46	2.63	42.68	33.18
2	420	3.29	67.90	60	245	26.67	0.14	0.20	5.00	3.65	42.88	35.03
2	365	2.18	63.35	65	250	13.33	0.16	0.20	5.46	2.27	35.04	30.23

## Results

**Fig. 4:**
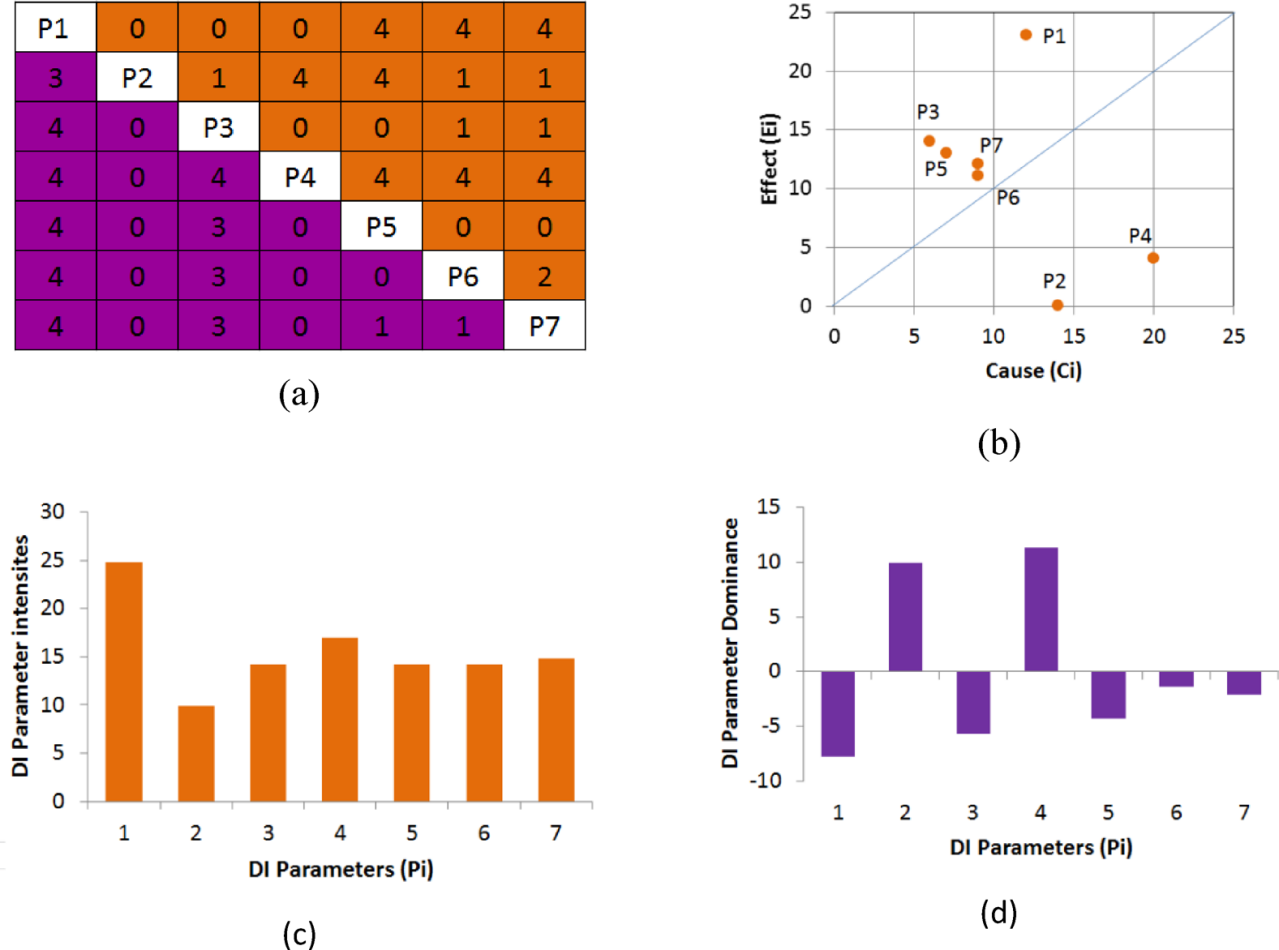
(**a**) The interaction matrix of the DI system, (**b**) Cause-Effect diagram, (**c**) Parameter interaction intensity plot, (**d**) Parameter dominance plot .

The DI system utilized the encoded database. First, the interaction matrix was composed based on the expert semi-quantitative method^42^ as mentioned in Sect. 4.2. This approach of encoding was shown to be good enough for evaluating mine dilution^60^. Next, the causes${C_i}$ and effects${E_i}$ were calculated according to Eq. (4); the parameters weights ${W_i}$ were determined using Eq. (5), and finally the DI values were computed according to Eq. (6). The interaction matrix, cause-effect graph, parameter interaction intensity, and parameter dominance diagrams are provided in Figs. 4(a-d), respectively. As can be seen in Fig. 4(b), the parameter constellation is almost perpendicular to the line E = C and little range in the parameter interaction intensity is observed (except for P1). This indicates that all 7 parameters used to establish the DI would be required, and none is redundant. The most interactive parameter and most dominant (see Fig. 4-d) in the system are P1 and P4, respectively. The least interactive parameter is P2. Similarly, Fig. 4(c)-c confirms that P1 and P4 are the top 2 important parameters. These results agree with the existing dilution charts and principles of stope overbreak and sloughing.

**Fig. 5:**
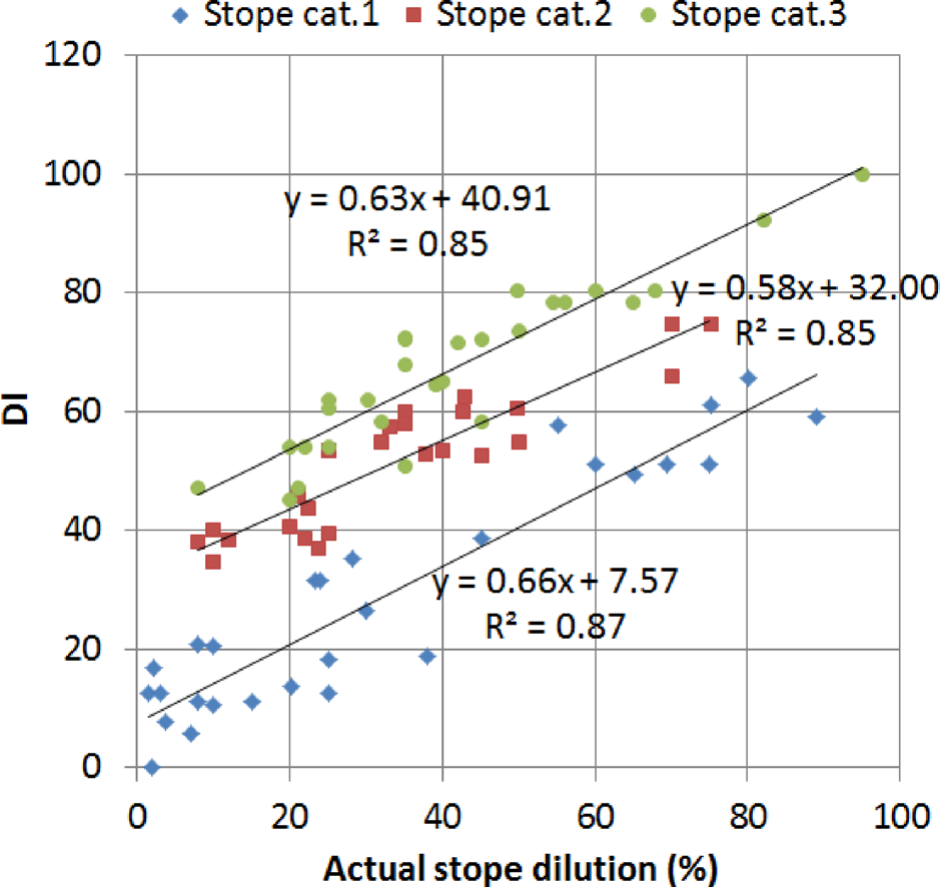
Actual dilution vs DI for each stope category.

**Fig. 6:**
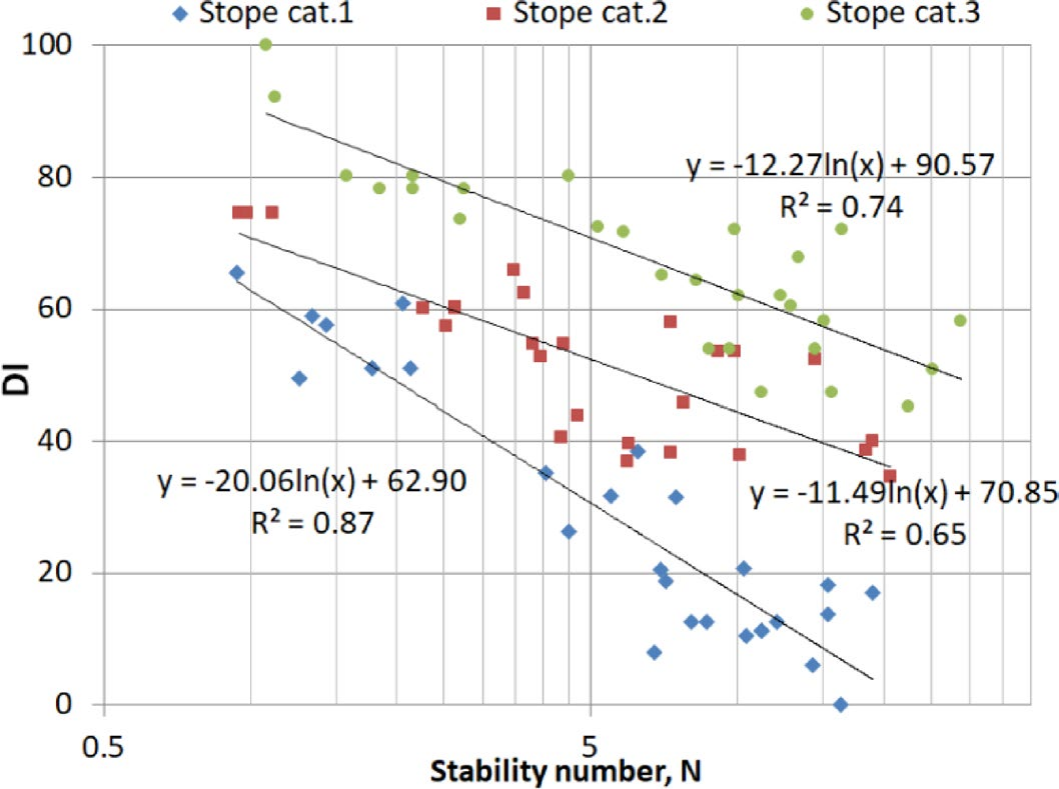
Stability number vs DI for each stope category.

**Fig. 7:**
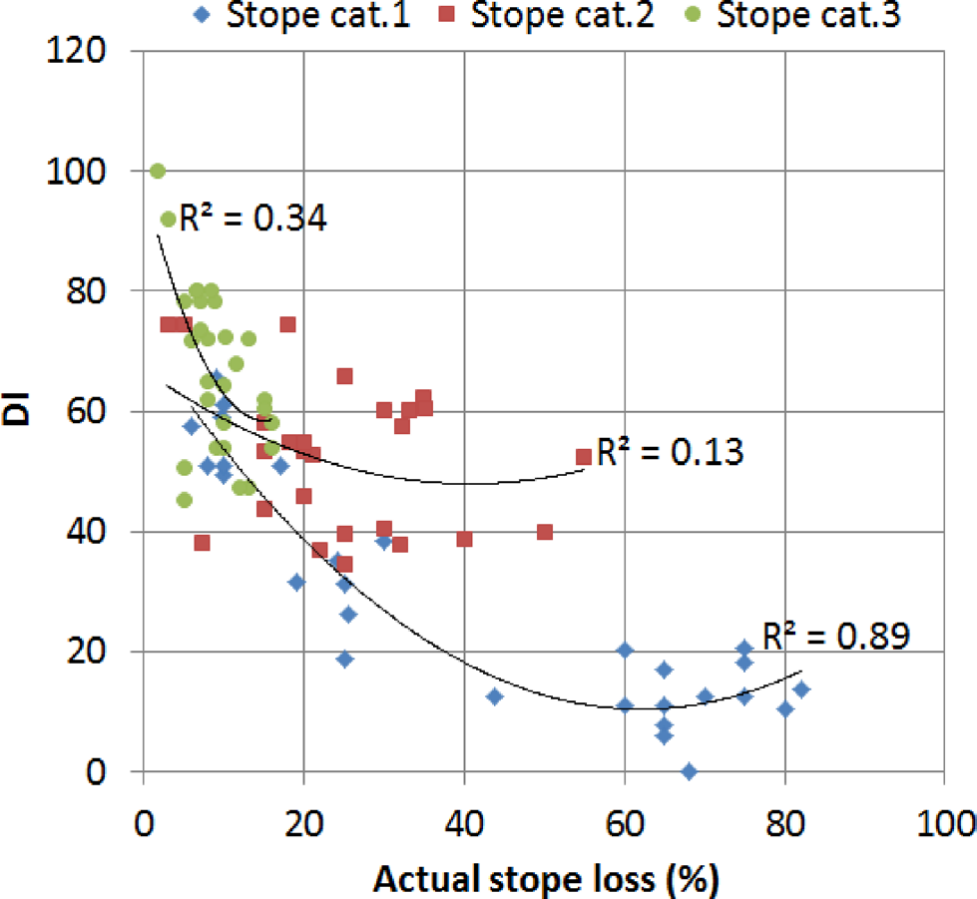
Actual stope loss vs DI for each stope category.

Table 5 provides a sample of the calculated DI. The DI values were compared to the actual dilution (Fig. 5), the stability number (N) (Fig. 6) and the stope loss (Fig. 7). Figure 5 indicated high correlations between the DI and the actual dilution (R^2^ > 85). This result implies that the DI can be used to estimate the dilution. In addition, the empirical correlations of DI vs. actual dilution for each stope category shown in Fig. 5 can be used to estimate the DI threshold that corresponds to a given stope category, as stope category relates well to the dilution levels, despite the overlapping of the data points per stope category. Overlapping mostly happens between categories 2 and 3 for the actual dilution between 20 and 40% (refer to Fig. 5). Despite this limitation, Fig. 5 shows that when the dilution is less than 20%, the simple shape yields the lowest dilution, the complex shape the highest, and the semi-complex a moderate dilution. Also, when the actual dilution is less than 20%, almost no overlapping of the data points is observed. If the acceptable dilution is set at 20%, the DI threshold can be estimated by plugging x = 20 into two equations (y = 0.63x + 40.9 and y = 0.58x + 32) shown in Fig. 5, which will give approximately DI = 40 and DI = 60 as the thresholds. Note that the 3rd equation (y = 0.66x + 7.57) is not used as it returns a very conservative value of DI for stope category 1. These thresholds also agree with the trends of DI vs. dilution (shown in Fig. 5); it can be seen that most data points for stope category 2 correspond to a DI of 60 in value. Therefore, any value exceeding this threshold is more likely to be associated with category 3. Similarly, most stope category 1 cases have DI values less than 40. In short, the proposed DI thresholds are 40 and 60.

**Table 5:** Sample of the calculated DI.

Discretized input parameters							DI	Actual dilution
P1	P2	P3	P4	P5	P6	P7		
0.1	0.6	0.2	0.8	0.6	0.2	0.6	26.36	29.89
0.1	0.8	0.4	1.0	0.8	0.2	0.8	58.98	88.91
0.1	0.8	0.4	0.8	0.8	0.2	0.8	51.07	69.25
0.1	0.8	0.4	0.4	0.8	0.2	0.8	35.26	28.09
0.1	1.0	0.6	0.2	0.8	0.2	0.6	31.63	23.27
0.1	0.4	0.4	0.8	0.4	0.2	0.2	7.91	3.63
0.1	0.8	0.4	0.4	0.8	0.3	0.6	31.44	23.97
0.1	1.0	0.4	0.2	0.8	0.2	0.2	12.52	1.51
0.1	0.6	0.2	0.4	0.6	0.2	0.6	10.54	10.01
0.1	0.6	0.2	0.6	0.4	0.2	0.6	12.52	3.00
0.5	0.6	0.4	0.8	0.8	0.2	0.4	52.80	37.73
0.5	0.6	0.2	0.8	0.8	0.2	0.8	60.05	42.68
0.5	0.8	0.4	0.4	0.8	0.2	1.0	62.36	42.88
0.5	0.8	0.4	0.8	0.8	0.2	0.4	57.41	33.14
0.5	0.6	0.2	1.0	0.8	0.6	0.2	60.38	49.84
0.5	0.4	0.6	0.8	0.8	0.2	0.4	54.78	32.02
0.5	0.4	0.6	0.2	0.6	0.2	0.8	38.30	11.89
0.5	0.2	0.4	0.8	0.6	0.2	0.4	36.99	23.77
0.5	0.6	0.4	1.0	0.8	0.2	0.8	74.55	70.04
0.5	0.6	0.4	1.0	0.8	0.2	0.8	74.55	75.05
1.0	0.8	0.4	0.8	0.8	0.2	0.6	78.25	65.02
1.0	0.8	0.6	0.8	0.6	0.2	0.6	78.25	54.56
1.0	0.8	0.4	0.8	0.8	0.2	0.8	78.25	56.01
1.0	0.6	0.4	1.0	0.8	0.2	0.8	80.23	67.78
1.0	0.6	0.4	0.8	1.0	0.2	0.6	80.23	60.05
1.0	0.6	0.8	0.8	0.6	0.2	0.6	80.23	49.72
1.0	0.8	0.6	0.4	0.8	0.2	0.2	72.42	35.04
1.0	0.2	1.0	0.8	0.2	0.2	0.8	64.42	39.17
1.0	0.8	0.6	1.0	0.8	0.2	0.8	92.13	82.01
1.0	0.8	0.4	0.4	0.6	0.3	0.8	61.94	30.10

Figure 6 indicates a very good correlation between DI and N for the stope shape category 1 (R^2^ = 0.87), while fair correlations were obtained for category 2 (R^2^ = 0.65) and category 3 (R^2^ = 0.74). The direct implication of this result is that N is also a good predictor of dilution for category 1 and category 3 to some extent; however, it does not correlate well with the DI in the case of stope category 2. This suggests that N doesn’t play a key role in the dilution in stope category 2; other factors such as operational conditions may contribute more, or perhaps stopes with semi-complex shapes could be considered as transient between simple-shaped and complex-shaped stopes. Figure 7 displays the correlation between DI and actual stope loss. Higher DI values correspond to lower values of the loss. Stope shape category 1 showed high correlation with the DI unlike the other categories.

**Fig. 8:**
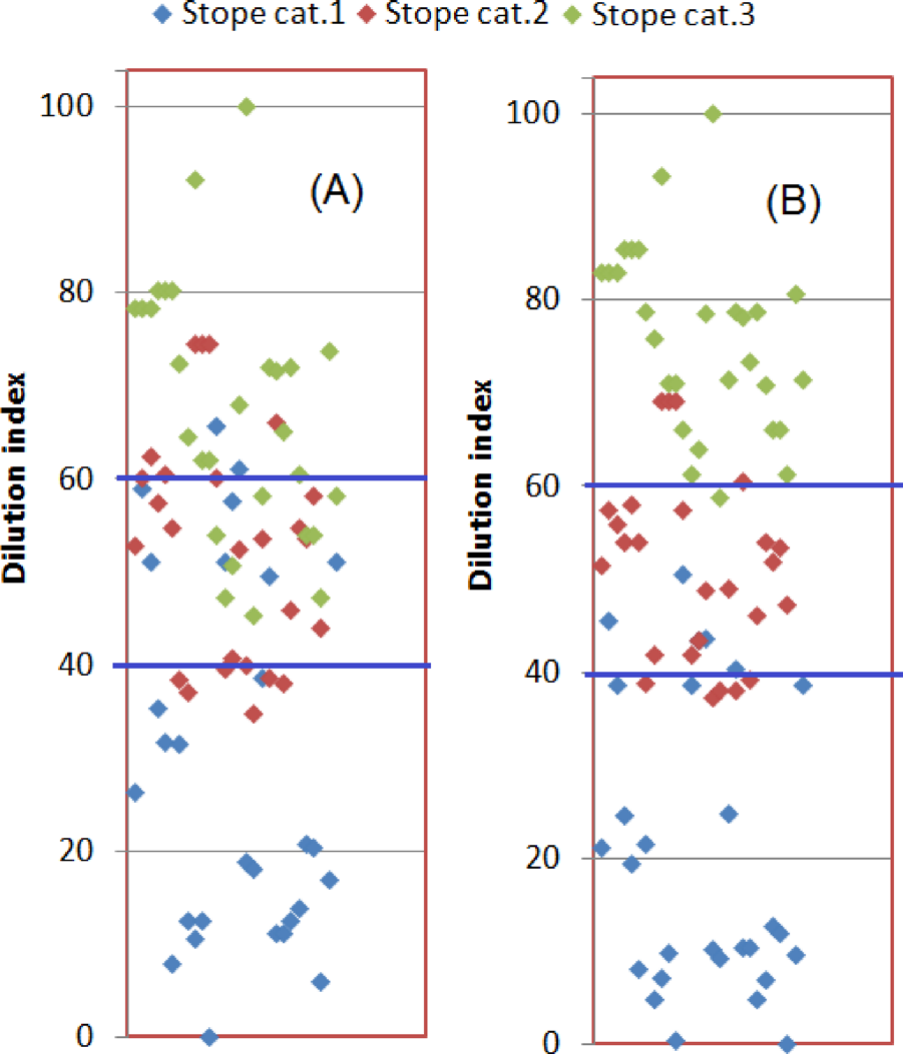
1D Dilution index diagram: (**A**) β=1; (**B**) β=2

The DI values were presented in the form of a 1D plot. Normalized values of DI were used so that the values are scaled within 0 to 100 (minimum value = 0 and maximum value = 100). Figures 8 (A-B) show the threshold values of DI that can be used to classify the dilution depending on the stope shape category. DI = 40 and DI = 60 are used to set the boundaries between the 3 categories (a DI less than 40 corresponds to stope category 1, DI ranging between 40 and 60 corresponds to stope category 2, and DI greater than 60 represents stope category 3). In Fig. 8 (A) and (B), β = 1 and β = 2 were used, respectively. It can be seen that Fig. 8 (B) indicates better classification. It should be noted that β values ranging from 0.5 to 4 were implemented, and the goodness of the DI and actual dilution fitting didn’t show any major difference; hence β = 2 was adopted as mentioned earlier. In short, the more complex the stope shape, the higher DI would be. This implies that high dilution and low stope loss would be expected. Conversely, low values of DI indicate low dilution, and a stope with simple shape irregularity is likely to be obtained; and most importantly, the stope loss can be estimated as well according to Fig. 7.

## Discussions

**Fig. 9:**
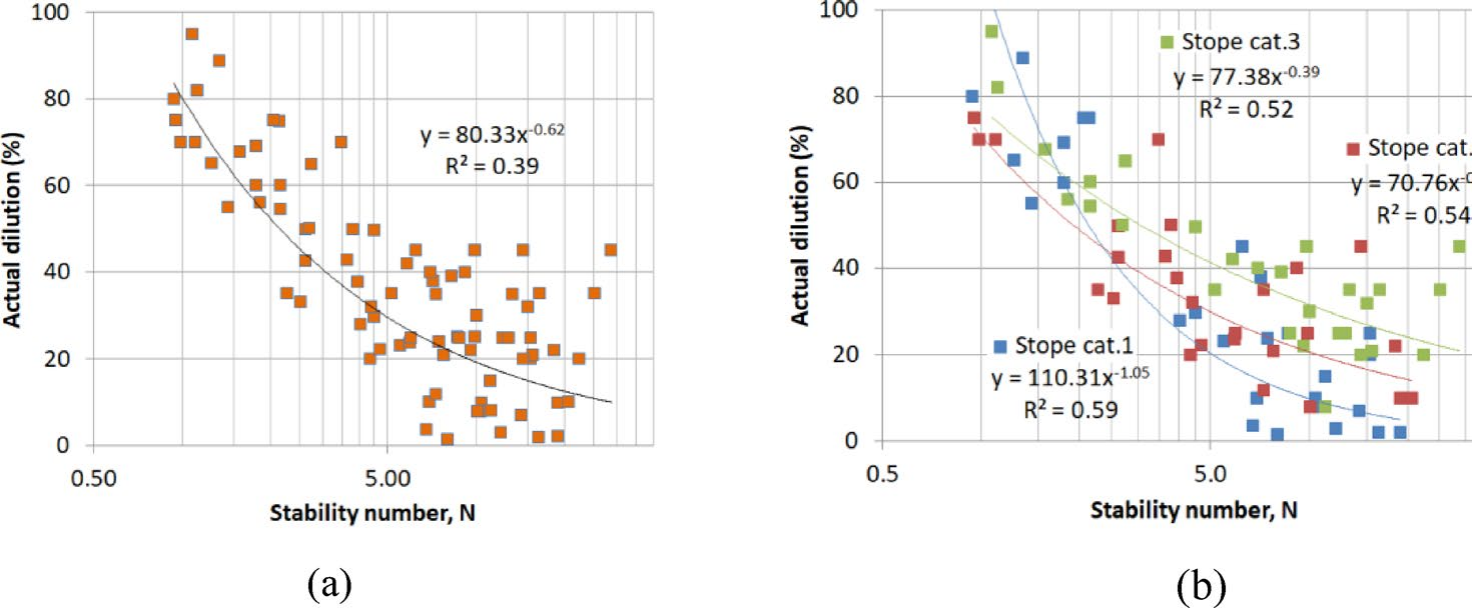
(**a**) N vs. actual dilution for all stope categories. (**b**) N vs. actual dilution for each stope category.

**Fig. 10:**
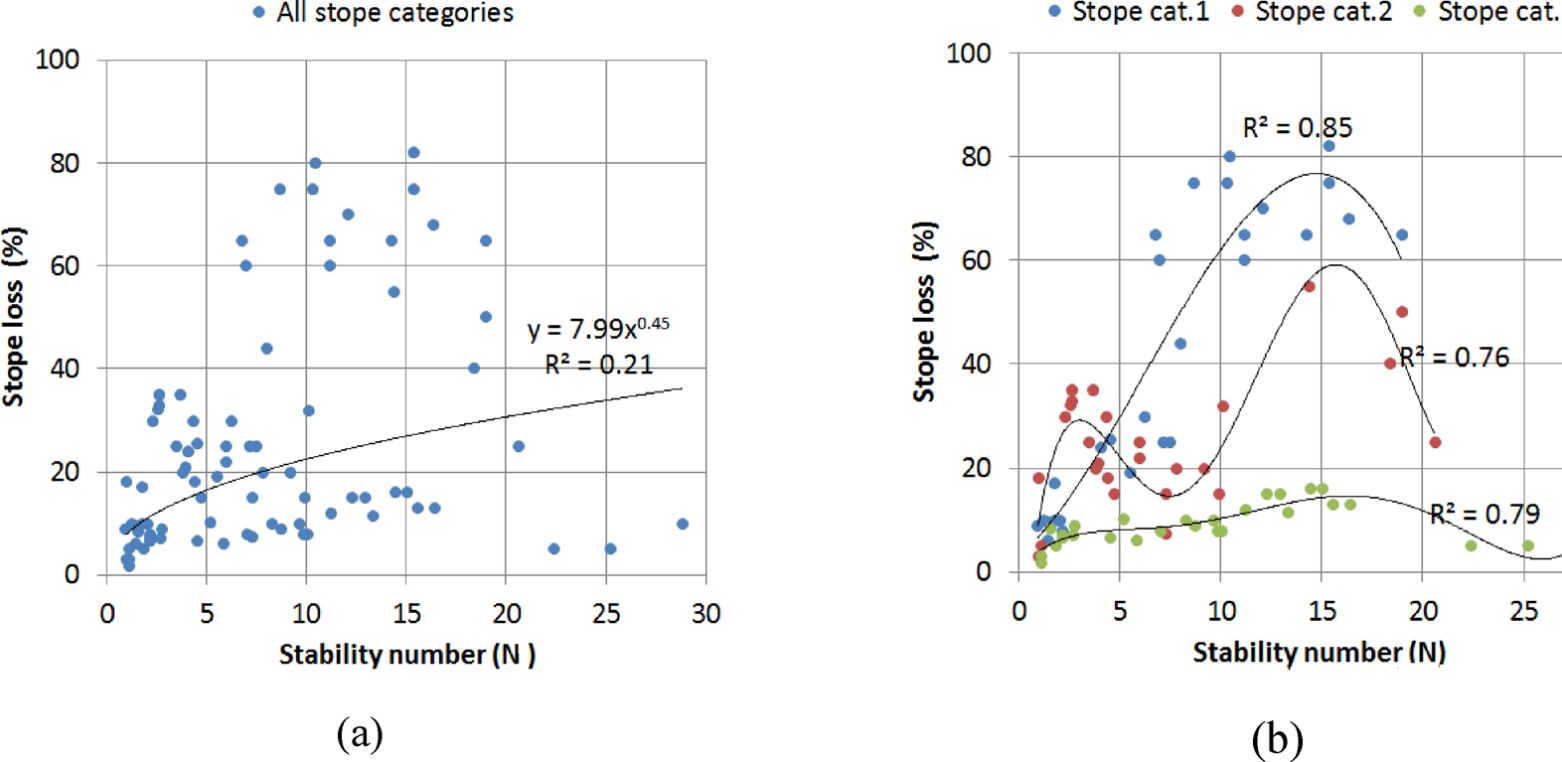
(**a**) N vs. actual stope loss for all stope category, (**b**) N vs. actual stope loss for each stope category.

**Fig. 11:**
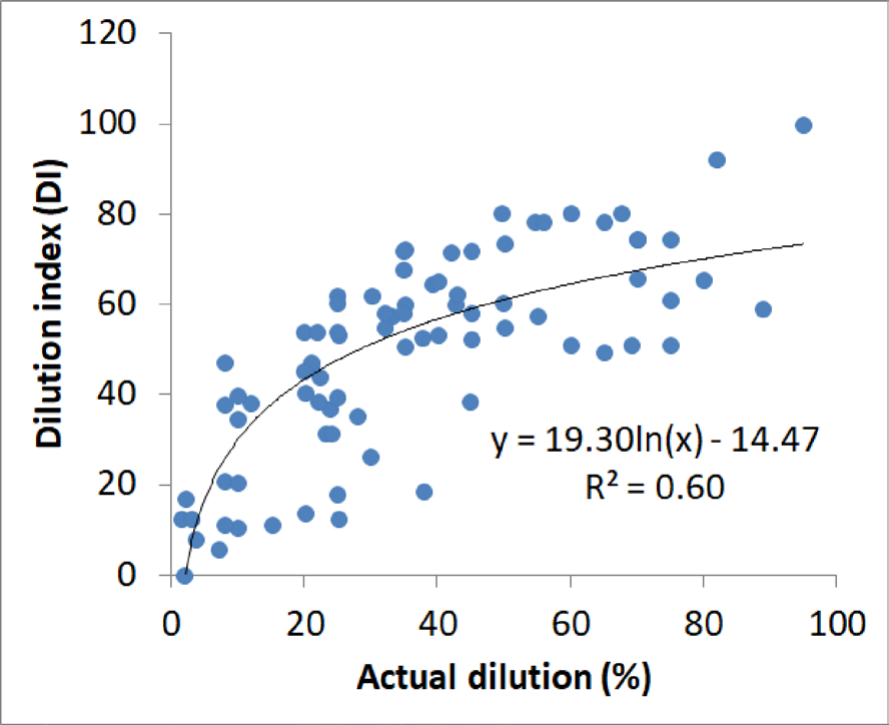
Actual dilution vs. Dilution index

**Fig. 12:**
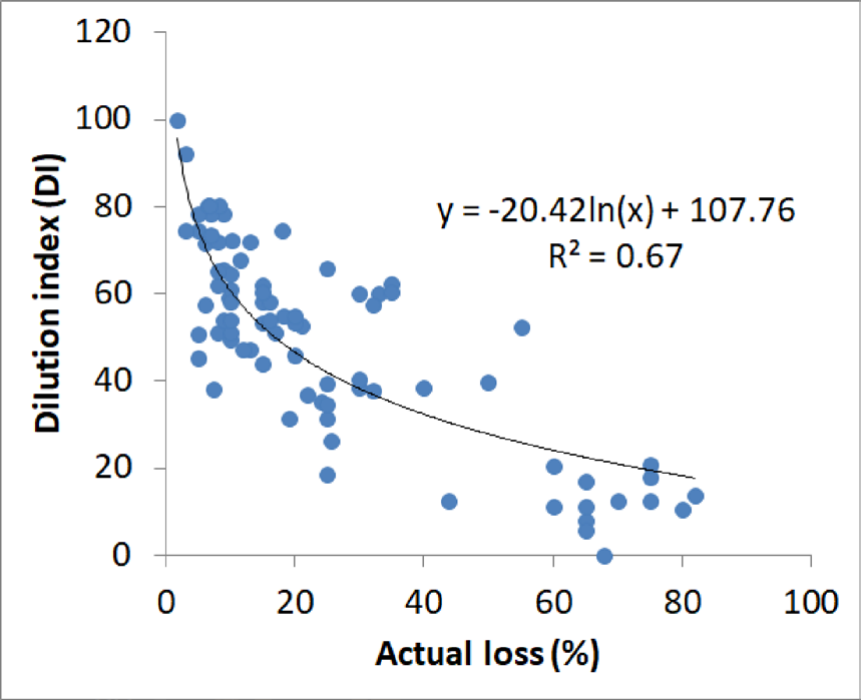
Actual stope loss vs. Dilution index

In the Ridder-Sokolny mine dilution database, the dilution level shows poor correlations with N (Figs. 9a-b) when the stope categories are not differentiated (R^2^ = 0.39 for the best possible fit). However, better correlations were obtained when the stope shape category was considered (R^2^ = 0.52–0.59). Similar results were achieved for the stope loss (Fig. 10a-b). This suggests that N, which is commonly used as a predictor for dilution^10^ cannot be employed to estimate the dilution in this study. This justifies the need for an alternative dilution predictor. On the other hand, the DI shows fairly good correlation with both the actual dilution and loss (Figs. 11 and 12). Hence, the DI can be used to estimate the stope dilution and loss. It should be noted that Fig. 10-b showing the stope loss fitting curves with several extrema. The stope loss was fit using polynomial expressions. This indicates that the stope loss is dependent on N but within certain ranges i.e., rock mass domains.

**Fig. 13:**
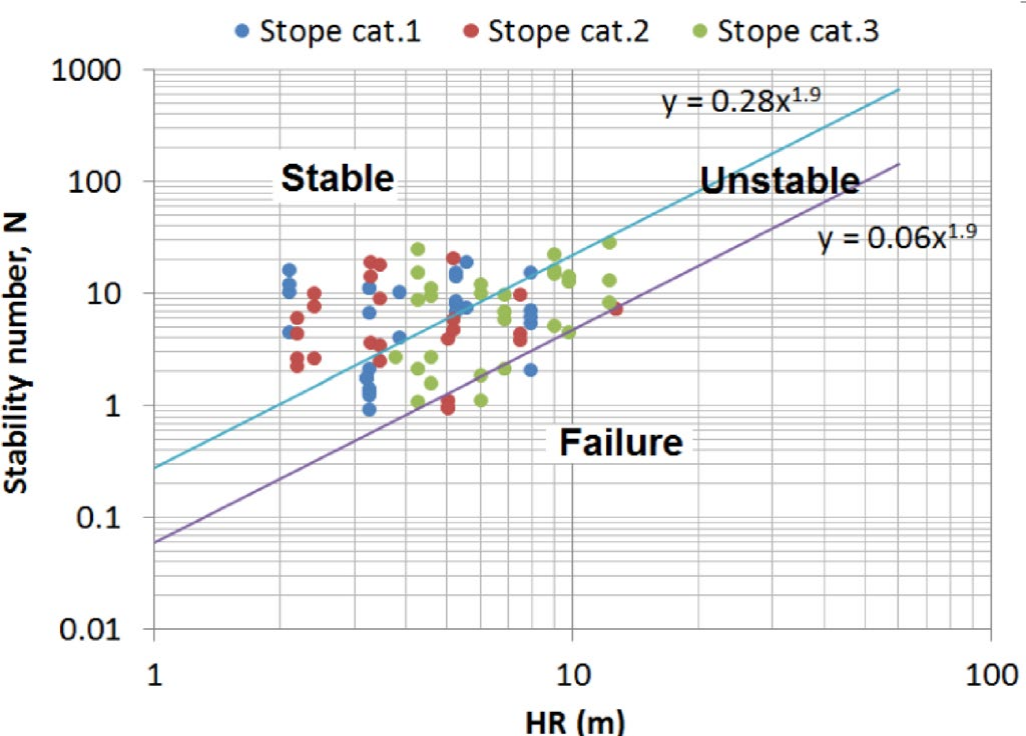
Stability graph of the stope surfaces

**Fig. 14:**
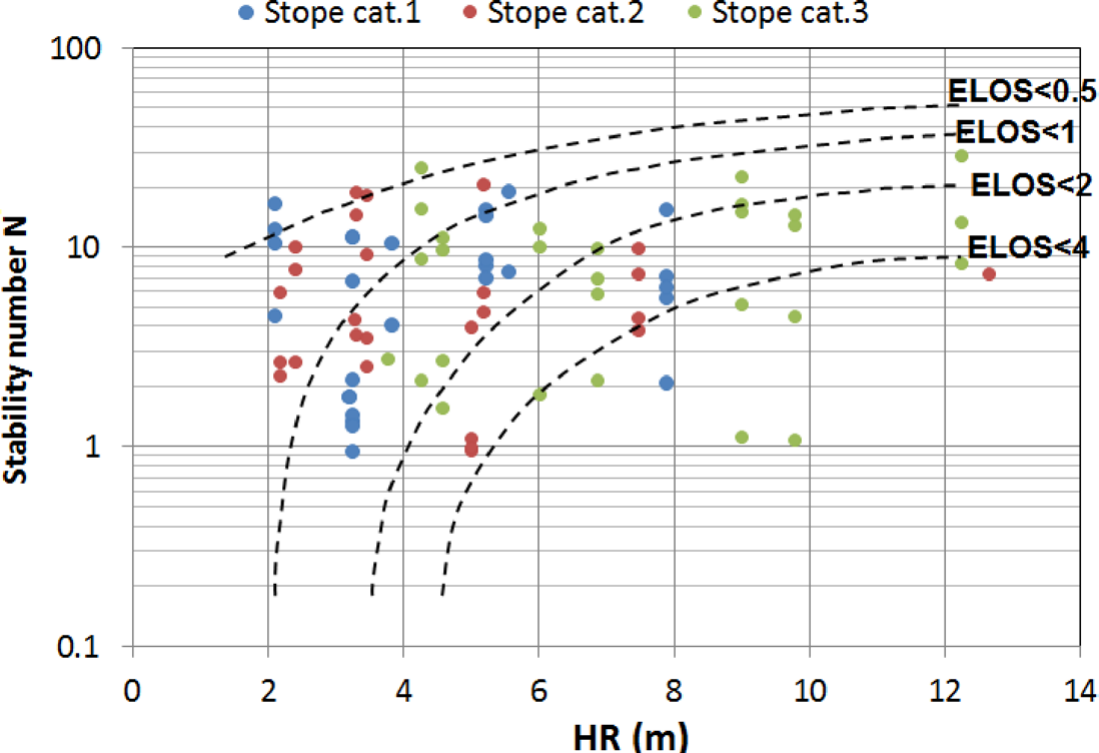
Equivalent linear overbreak/sloughing (ELOS) graph for this study

In addition, the results are compared with the stability graph^56^ and the dilution graph^13^. The data of the present study are superimposed on these graphs. It can be seen in Fig. 13, the data points fall within stable and unstable zones, which is in agreement with the field observations. There was no major failure of the stope walls investigated. It can also be seen that most stopes with complex shape irregularity are found in the unstable zone of the graph, while most of the stope with simple and semi-complex shapes seem to be within the stable zone. In spite of this fair consistency, the stability graph falls short in adequately accounting for the shape irregularities. In Fig. 14 the dilution graph is provided. Once again, the data points appear to disperse randomly across the ELOS graph. This indicates that there is no consistent relationship between the ELOS and the stope shape irregularity. In summary, the DI proposed comes in very handy when the stope shape irregularity has to be considered and for situations when N is not a good predictor of the stope performance.

**Fig. 15:**
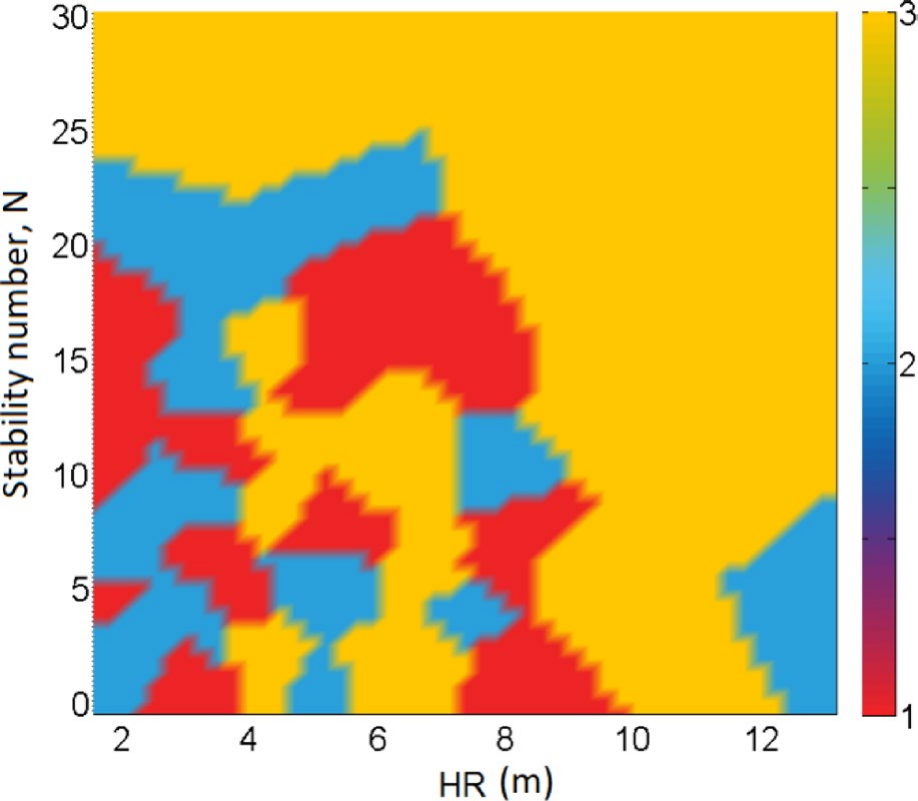
2D Stope category distribution map.

The factors leading to the complexity of the stope shape, such as poor blasting, uncertainties in the geological block modelling, and other operational conditions, are not investigated at this moment and are beyond the scope of this study. However, it is important to look into the distribution of the stope categories. To this end, a 2D distribution map is provided in Fig. 15. As can be seen, it is quite difficult to associate the occurrence of the shape with any particular values of N and HR. Nevertheless, simple shape seems to be associated with HR < 10 and N20; semi-complex shape mostly corresponds to HR between 2 and 4 and N less than 25; complex shape, however, is associated with HR between 4 and 7 and between 10 and 12. This map indicates that the stope shape is more likely to be dictated by the HR values than N. The HR depends on the ore body geometry and the employed mining method.

**Fig. 16:**
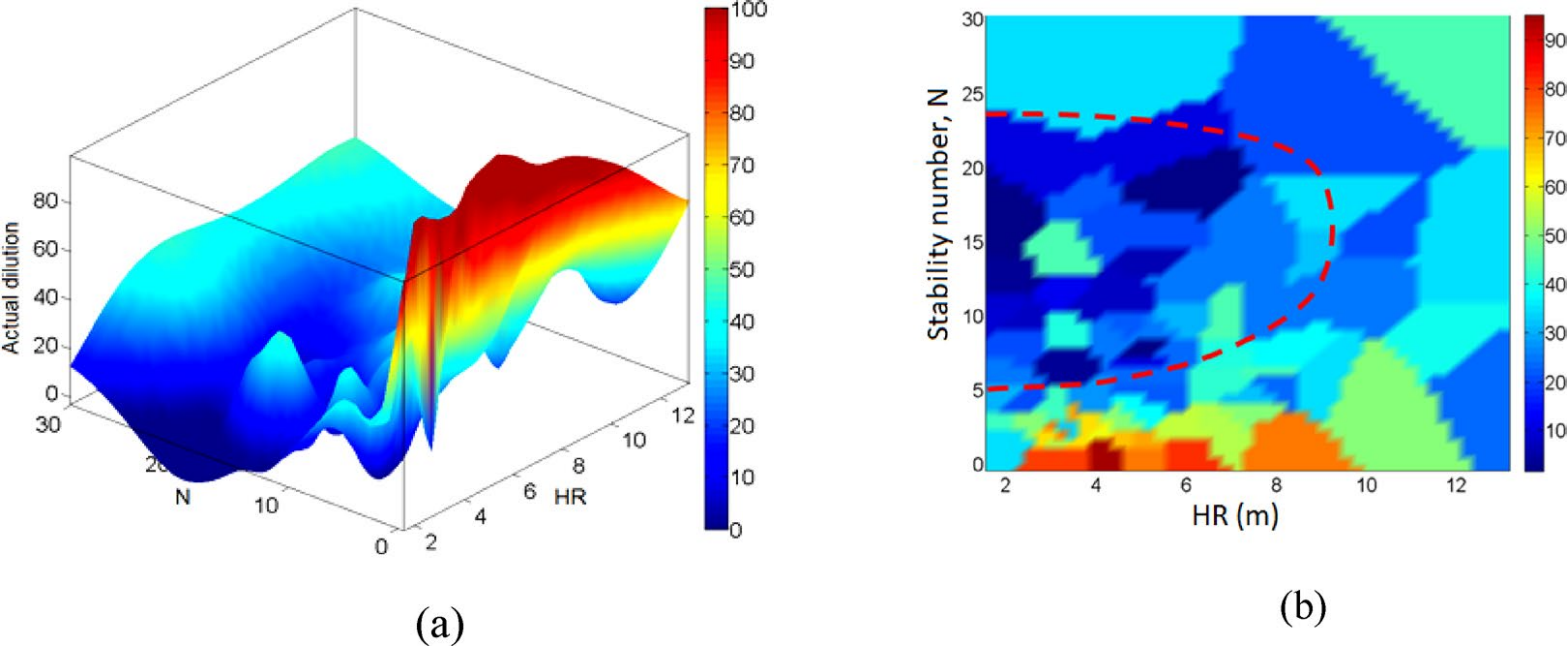
(**a**) 3D Actual dilution map. (**b**) 2D Actual dilution map

**Fig. 17:**
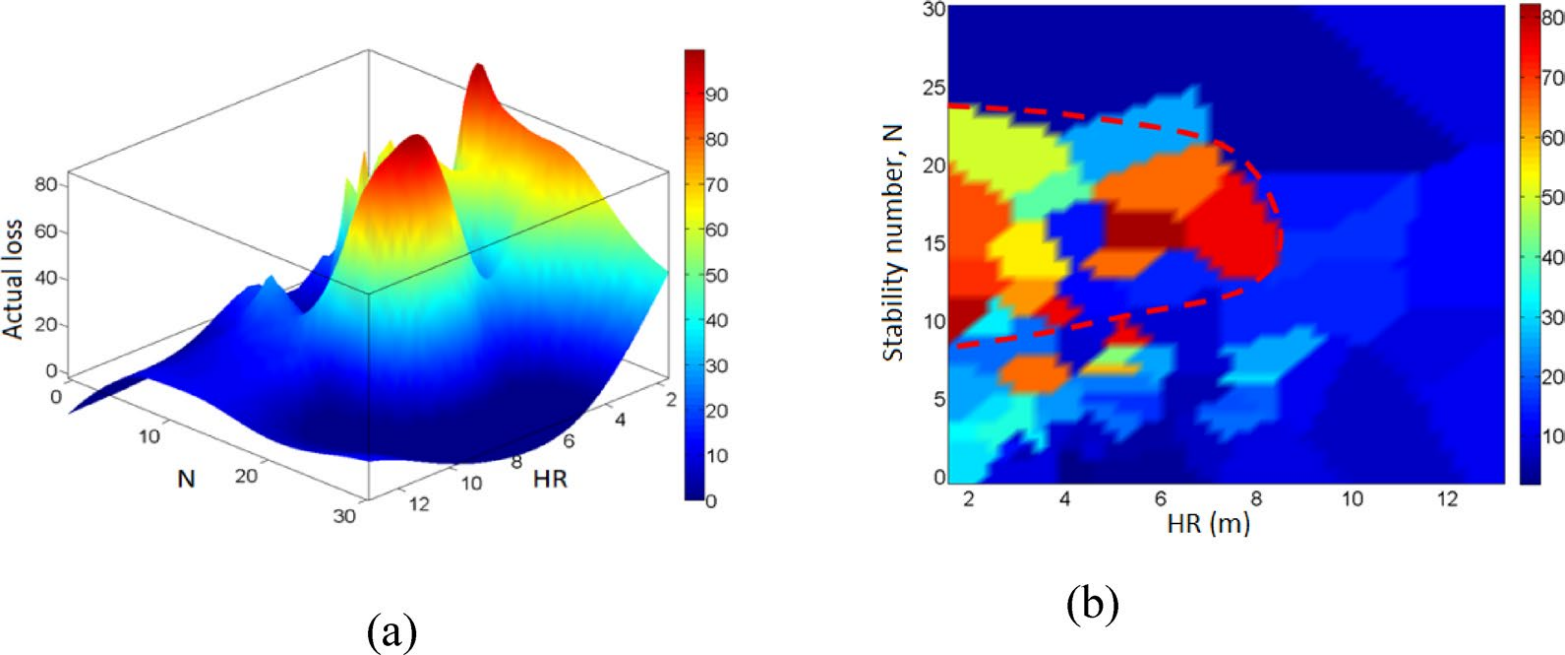
(**a**) Actual stope loss map. (**b**) Actual stope loss map.

In comparison with the actual dilution (Fig. 16(a-b)) and actual stope loss (Fig. 17(a-b)), N seems to play a key role. Lower dilution is associated with specific domains of N and HR, as highlighted in Fig. 16-b, i.e. 5 < *N* < 25 and HR < 9. Meanwhile higher dilution is related to *N* < 5 and HR > 2; this relationship is in agreement with the principle of dilution and sloughing. Similarly, higher stope loss is related to certain rock domains and stope dimensions, as illustrated by the highlighted area in Fig. 17(b). This area of higher loss matches with the area of lower dilution in Fig. 16(b). By superimposing Fig. 15 on either Fig. 16-b or Fig. 17-b, it can be seen that stope shape complexity is quite independent of the dilution and loss, which makes it even more difficult to quantify the effect of the shape irregularity by using existing tools. This is precisely one of the main contributions of this work. Note that Figs. 15, 16 and 17 were plotted using the curve fitting toolbox in MATLAB software, version 2024b, which allowed us to fit surfaces/maps to the compiled stope performance data via interpolation.

The greatest benefit the DI method can provide is not necessarily to directly estimate the stope dilution from the DI graphs, but rather to assist the designer to examine the effect of the stope shape irregularity on the dilution level in a systematic manner, which would allow for a logical adjustment of the design when necessary. This exercise can be combined with other empirical design tool methods, numerical analyses, and production and economic requirements in order to achieve an optimal design of the stope to minimize dilution and loss. Hence, the DI method is one of the several stope design tools in connection with dilution that have been developed so far and could be used when needed. It is not intended to use the DI as a substitute for the existing empirical methods. The methodology can be adopted at any site with specific conditions.

When implementing the DI method with new data, it is very important to avoid believing that the method is a very rigorous analysis. Non-rigorous methods are the basic foundation of empirical design tools for mine stope^10,20^. Users should not rely on the accuracy of the DI threshold to decide on the level of expected dilution. The best use of the method will be to derive one’s own DI as data become more available. A practical use of the DI method during the early stage of the design is to select the input parameters for economic reasons along with intuitive reasons whenever necessary and to ascertain where they plot on the DI graph. Next, for outlier data points, it is advisable to carefully re-examine the input data to answer why the data does not fit the graph. This might be more valuable since it would lead to a back analysis along with sound engineering judgment. Any particularity of the design, for instance, the presence of unfavorable operational conditions and geological features (major faults, significant inclusions of rocks of differing strength, etc.), can then be considered on a case-by-case design basis. It is worth mentioning that, despite the merits of the DI methodology, there are a number of limitations. For example, the DI threshold could have been determined in a more objective manner. Since the data size was limited, the determination of the thresholds was done by analyzing the trends of the graph of DI vs. actual dilution. Further study could employ a probabilistic approach to determine the stope shape category that is most likely associated with a given value of the DI, assuming a normal distribution of the DI values, to be able to ultimately identify the threshold. In summary, the results of this study can be employed for stope design analysis in connection with minimizing dilution in two contexts. For the design of new stopes in the Ridder-Sokolny mine operations, the results can be directly applied: (1) Rate the input parameters according to Table 4 and the instructions given in Sect. 5.2; (2) calculate DI using Eq. (6); and (3) use the empirical equations provided in Figs. 5, 11 and 12 to estimate the DI threshold and the unplanned dilution, respectively.

If the methodology is applied to a new mine site with different geo-engineering settings, an initial DI system should be developed using data specific to that particular mine. However, the steps to follow remain the same, and they are highlighted as follows:

- Step 1: Compile a dilution dataset;
- Step 2: Select the input parameters, including those related to the stope geometry and rock mass properties commonly used in the empirical stability graph methods;
- Step 3: Rate the selected input parameters according to Table 4 and the instructions given in "Parameters selection and rating"
- Step 4: Establish the RES interaction matrix using the semi-quantitative approach as suggested in this research (refer to Fig. 4a);
- Step 5: Calculate the causes, effects, weights of the parameters, and the dilution system index as defined in Eqs. (4–6), respectively;
- Step 6: Determine the dilution thresholds by plotting the DI vs. actual dilution graphs for each stope category (refer to Fig. 5). Use the acceptable dilution in the mine (e.g., 20%) to find the thresholds as detailed in Sect. 5.3;
- Step 7: Produce the DI graph (refer to Fig. 8) and use it to estimate the performance of the stope given the geometrical and rock mass conditions. The DI can also be used in an interactive parametric study to determine the HR (hence, the stope width and length) that minimizes the dilution given the stope shape category.

## Conclusions

This paper presents a methodology in which the effect of the stope shape irregularity on unplanned dilution and ore loss can be quantified based on available mine design and geotechnical data. Firstly, the numerical modeling approach was used to illustrate the effect, and the results indicated that the more complex the stope shape, the higher the sloughing and dilution. Next, the RES was employed to develop the DI to quantify the effect. The results revealed that the stope category had the most significant effect on the DI. Overall, the DI showed a good correlation with the actual dilution and stope loss. The results showed that the DI is a better predictor of dilution compared with the stability number (N) based on the data employed. Also, the present study showed that the unplanned dilutions were inconsistent with some of the existing empirical graphs. Therefore, the results of this study constitute a reasonable solution for the current need to incorporate the stope shape irregularity effect in dilution assessment.

The present study has some limitations, as do any empirical methods. Further studies could investigate ways to determine more objectively the DI threshold values used to differentiate the stope shape irregularity. The results presented here are based on “educated guesses,” the authors’ experience, and the available data. However, by following the important steps outlined, the approach used in this study can be applied to mining operations with additional factors that affect unplanned dilution. It is important to remember that the DI method is non-rigorous, similar to the stability graph. Emphasis should not be put on whether a new data point would fit within the defined threshold or not; instead the user should examine the reasons for the particular situation and make appropriate decisions. The strength of the present method relies heavily on the data base from which it is derived, adding more data to the current database will further improve the method’s predictive ability. Generally, the results of the present study showed some merits over existing results. It is concluded that the DI could be an alternative tool for unplanned dilution assessment as it implicitly accounts for operational conditions and the orebody complexity.

## Data Availability

The data generated and analyzed during the current study are available from the corresponding author upon reasonable request.
